# A case study of a new dyslexia course created for university teacher preparation programs

**DOI:** 10.1007/s11881-025-00341-2

**Published:** 2025-09-24

**Authors:** Mitzi Berryhill, Kayla Reggio, Kim Skinner, Laura Piestrzynski, Estanislado S. Barrera IV

**Affiliations:** 1 AIM Institute for Research and Learning, Conshohocken, PA USA; 2 Lighthouse Academy for Dyslexia, Ocean Springs, MS USA; 3 Lutrill & Pearl Payne School of Education, Louisiana State University, Baton Rouge, LA USA

**Keywords:** Dyslexia, Dyslexia course, Literacy legislation, Preservice teachers, State mandates, Teacher preparation programs

## Abstract

In an effort to increase the dyslexia knowledge of future classroom teachers, in 2022, the state of Louisiana passed Act 607, which requires all students seeking Louisiana teacher certification to complete a three-credit hour course specific to dyslexia starting in the 2024–2025 school year. With the implementation of the inaugural dyslexia courses in Fall 2024, this study examined the experience of creating and implementing this new course from the perspectives of the instructors and the teacher education students at a public state university. Utilizing an embedded case study approach, data sources included the course syllabus, course instructional materials, student surveys and reflections, and instructor analytic notes and reflections. The findings reveal the surprises, successes, and obstacles experienced by participants in this newly required course, offering practical insights for adapting legislative mandates into effective educational practices. This study provides a framework for universities nationwide to enhance literacy instruction within teacher preparation programs, contributing to the broader effort of improving outcomes for students with dyslexia.

## Introduction

Estimates of dyslexia prevalence vary, with figures ranging from 7 to 20%, influenced by differences in definitions and diagnostic practices (Hall et al., 2023; Yale Center for Dyslexia & Creativity, 2022). Peltier et al. (2020) state that around 34% of students receiving special education have a diagnosis of specific learning disability or SLD, and over 80% of students with an SLD are experiencing reading difficulties. While the prevalence varies, it remains a common reading disorder that can significantly impact reading ability and progress in school.

With dyslexia research and advocacy continuing to grow, legislation is being formed to support the identification and remediation of dyslexia in schools (Gerain et al., 2020). Currently, 49 of 50 states have some type of legislation that addresses dyslexia, such as screening mandates for dyslexia or intervention requirements (National Center for Improving Literacy, 2024). These laws vary widely in scope, with some focusing solely on early screening and intervention and others encompassing professional development and comprehensive support systems (Gonzalez & Brown, 2019). Additionally, fourteen states have recent legislation that includes requirements for preservice teachers, designed to increase dyslexia understanding in teacher preparation programs (National Center on Improving Literacy, 2024). Legislation mandates have a large impact on practices, so it is important to review the legislation and its effectiveness.

## What dyslexia is and what it is not

In 2002, the International Dyslexia Association developed a definition of dyslexia to address the current research and misconceptions around dyslexia. The definition states the following:

Dyslexia is a specific learning disability that is neurobiological in origin. It is characterized by difficulties with accurate and/or fluent word recognition and by poor spelling and decoding abilities. These difficulties typically result from a deficit in the phonological component of language that is often unexpected in relation to other cognitive abilities and the provision of effective classroom instruction. Secondary consequences may include problems in reading comprehension and reduced reading experience that can impede the growth of vocabulary and background knowledge.

(para. 1)

As the term dyslexia has been around for decades, this definition served as an overview of what the term dyslexia encompasses, which was and continues to be imperative to the dyslexia community as misconceptions around dyslexia have been revealed and refuted over time.

While this definition of dyslexia remains one of the most widely used definitions today, it is worth noting that many researchers in the field of dyslexia suggest that a new definition of dyslexia may be warranted. Odegard et al. (2024) state, “Over the past two decades, the IDA definition has shaped how dyslexia is understood and addressed across multiple contexts. However, advancements in scientific knowledge, changes in educational practices, and societal expectations necessitate a critical review of this foundational definition” (pp. 279–280). The definition of dyslexia is complex as it works to serve a variety of fields and a continuum of students.

Despite the development of a consensus around the definition of dyslexia, many misconceptions regarding dyslexia remain. Washburn et al. (2016) suggest that the condition’s core phonological deficits continue to be misunderstood among educators. Historically, dyslexia was often misinterpreted as a visual processing issue which results in reading and writing letters backwards. Vision therapy, colored lenses, or color overlays were suggested for dyslexia in the past in connection to the idea that vision issues were the cause of dyslexia, but research has concluded that these are unnecessary and not evidence-based approaches for dyslexia as it is a language disorder instead of a vision disorder (Gonzalez, 2021; Peltier et al., 2020; Shaywitz & Shaywitz, 2020). Other misconceptions include dyslexia as an indicator of low intelligence and the idea that dyslexia is not a lifelong condition (Kirby, 2020). It is imperative to address these misconceptions in teacher education programs to ensure that students with dyslexia receive appropriate remediation and classroom support.

## Teacher knowledge of dyslexia

Teacher preparation programs are frequently criticized for falling short in attempts to adequately teach the characteristics of dyslexia and effective instructional practices for students with dyslexia, leaving educators underprepared to meet the needs of students with reading disabilities (Washburn et al., 2011, 2016). Educator knowledge of dyslexia has been researched among classroom teachers as well as participants in teacher preparation programs. Gonzalez and Brown (2019) suggest that early childhood educators and centers also need training around dyslexia to support early identification. Gonzalez (2021) points out that multiple studies have found misconceptions around dyslexia are prevalent among students in teacher preparation programs and misconceptions remained in in-service educators who lacked dyslexia-focused training. Knight (2017) found that additional training significantly enhanced teachers’ confidence and ability to support students with dyslexia. With a high prevalence of dyslexia in the classroom and misconceptions still remaining concerning what dyslexia is, it is important that teacher preparation programs focus on this reading disorder to provide preservice teachers with a clear definition and overview of dyslexia.

## Adult learning theory

This case study was informed by Knowles’ (1978) adult learning theory, which emphasizes the unique needs and characteristics of adult learners. Key elements of this theory include the learner’s self-direction, the use of prior experiences as a foundation for new learning, the need for learning to be relevant and immediately applicable, and a preference for problem-solving over rote memorization (Knowles, et al., 2014). Self-directed learning in the classroom refers to instructors creating opportunities for students to take some ownership in what they are learning instead of simply being recipients of the learning objectives outlined by the instructor. As the instructors of the courses in this case study work frequently with adult learners who have varied experiences with dyslexia, this framework guided much of the preparation for this inaugural course.

While the dyslexia course was mandated for preservice teachers by legislation, as dyslexia advocates in the community, the instructors recognized that meaningful conversations and intentional course design were essential to ensuring students in the teacher preparation program had opportunities to engage in learning that was both relevant and practical for their current and future experiences. By incorporating collaborative projects, such as the jigsaw presentations, and assignments directly tied to their future teaching roles, the dyslexia course leveraged students’ existing knowledge while fostering new understanding in meaningful contexts. Additionally, adult learning theory (Knowles, 1978; Knowles et al., 2014) provided a framework for analyzing final course surveys and feedback, guiding the evaluation of what elements students considered successful, and identifying areas needing refinement. This theoretical lens helped contextualize student responses and shaped recommendations for improving the course in subsequent offerings.

## Requirements and implementation of Louisiana’s Act 607

In keeping with the dyslexia policy agenda (Gearin, 2020), House Bill No. 136 became Act No. 607 in 2022 during Louisiana’s regular legislative session, thus requiring teacher preparation programs to include a portion of their required literacy coursework dedicated to a three-credit hour course focused on teaching students with dyslexia beginning with the 2024–2025 academic year. Specifically, Act 607 (Louisiana Legislature, 2022) required the new course to provide a scientifically grounded overview of the history, epidemiology, and clinical presentation—early indicators and common and persistent indicators seen in classrooms. The course must also provide an overview of evidence-based instruction, remediation, fortification, and accommodations. Additionally, Act 607 (Louisiana Legislature, 2022) indicates that the new course provides teacher candidates with information on how to become a dyslexia practitioner or dyslexia therapist.

Concerning the implementation of the new course, Act 607 (Louisiana Legislature, 2022) indicated that the new course had to be added to current degree plans within the existing hours and that “each program shall designate at least one faculty member to teach the coursework who has been provided specialized training in instructing future teachers on how to teach students with dyslexia” (p. 2). Aware of these requirements, we began the review process outlined by our university’s Faculty Senate Courses and Curricula policy statement on creating a new course. We also had to inform all of the teacher preparation programs in our college—early childhood education (birth to K and K-5), elementary grades 1–5, dual certification (elementary grades 1–5 and special education 1–5), and master of art in teaching for elementary and the master of art in teaching for secondary—as well as several others in the College of Agriculture, College of Humanities and Social Sciences (English, history, French, and Spanish), College of Music (vocal and instrumental), and College of Science (math and science) about the required new course so that program curricula could be revised to include the new dyslexia course.

Once the new course was approved and program curricula were revised, the next step in implementing Act 607 was to begin offering the course. Although transcripts would not be audited for the new course until May 2028 to qualify for certification, we felt it was important to provide our teacher candidates and residents with a course addressing dyslexia as soon as possible; therefore, the inaugural offering of the course was in the fall of 2024, and students were given the option to take the new dyslexia course as an approved substitution. Teacher candidates in the early childhood and elementary grades programs could substitute an arts integration course, and candidates in secondary programs could substitute a content area reading course with the new dyslexia course.

## Methodology

This study utilized an embedded case study design (Yin, 2018) to examine the instructors’ process of designing and implementing and the students’ outcomes from participation in an inaugural, state-mandated dyslexia course for preservice teachers in response to Louisiana’s Act 607. Yin (2009) explains a case study as understanding a phenomenon in its real-world context. The research focused on understanding the experiences of the instructors and students involved in the course, analyzing the course syllabus and materials, reflections, and survey feedback to identify successes, challenges, and implications for future implementation.

This research addressed the following questions:

1. What were the successes and challenges discovered by instructors while designing and implementing a state-mandated dyslexia course for students enrolled in Louisiana teacher preparation programs?
2. How did participation in the undergraduate dyslexia course impact preservice teachers’ knowledge of dyslexia?

This study details how the legislative mandates were put into action through the course creation and instruction and provides practical considerations for other teacher preparation programs.

### Participants and setting

The participants were 40 preservice teachers enrolled in one of two sections of the new dyslexia course at a large state university. In the first section, 11 of 11 participants were females. Of the total participants, nine were enrolled in the dual certification program to receive teaching licenses in elementary education as well as special education. In the second section, 27 of 29 participants were females, with two male participants. Of the total participants, thirteen were enrolled in the dual certification program to receive teaching licenses in elementary education as well as special education. The other participants were elementary education preservice teachers. The participants were in the fall semester of their junior year. Participation in the study was voluntary, and informed consent was obtained from all participants prior to data collection.

### Course development

The Louisiana Board of Regents partnered with a local dyslexia training center to support the development and implementation of the law. A standard course syllabus was drafted and created by one of the authors, a certified academic language therapist–qualified instructor (CALT-QI). The course syllabus was created to meet the specifications of the law. The sample syllabus included course textbook selections, example course readings, an example course description, course objectives, and 16-week overview. Each week was outlined, including the topic, objectives, description, and example assessment of learning. Subject matter expertise and prior knowledge and experience from professional dyslexia training were utilized to curate the syllabus. In response to the need for creation of sample syllabi, the Louisiana Board of Regents hosted a 2-day Dyslexia Symposium to assist faculty at state universities with the preparation and logistics of course development. The symposium included basic training in the history, epidemiology, and evidence available regarding dyslexia, group workshopping to review materials that outline evidence-based practices, and the distribution of the sample course syllabus. The sample course syllabus was developed so that instructors without knowledge and experience teaching dyslexic students could still successfully implement the course given the appropriate materials.

The sample syllabus provided became the skeleton for the course offered. Using the sample syllabus, two literacy education professors collaborated to make edits and add professional input based on additional required coursework in the department and to ensure the syllabus reflected a well-rounded approach to dyslexia and literacy instruction. The edits included the addition of appropriate university policies and requirements to the syllabus.

Once instructors for the course were assigned, the two instructors for the course met to discuss the requirements of the law and the proposed outline provided by the Board of Regents and made further adjustments prior to implementation. Some of the adjustments included the development of reflection questions for students, the addition of materials newly released by the Louisiana Department of Education, and sharing ideas concerning specific assignments.

### Course materials and assignments

The course syllabus describes how the course explores an overview of the body of scientific work regarding dyslexia, beginning with its historical roots up through present-day findings. Practical applications of common ways to identify those who may be at risk for dyslexia, including early and persistent indicators, as well as common classroom presentations, are discussed.

The required readings listed in the syllabus for the course were as follows:

Required texts:

Birsh, J. R., & Carreker, S. (Eds.). (2018). *Multisensory teaching of basic language skills* (4th ed.). Brookes. ISBN-13: 978-1681252261

Shaywitz, S. E., & Shaywitz, J. (2020). *Overcoming dyslexia* (2nd ed.). Vintage. ISBN-13: 978-0679781592

Additional readings:

Ferrer, E., Shaywitz, B. A., Holahan, J. M., Marchione, K. E., Michaels, R., & Shaywitz, S. E. (2015). Achievement gap in reading is present as early as first grade and persists through adolescence. *Journal of Pediatrics, 167*(5), 1121–1125. 10.1016/j.jpeds.2015.07.045

The Yale Center for Dyslexia and Creativity. (n.d.). *Speaking with one voice: A guide to talking about dyslexia.* https://dyslexia.yale.edu/wp-content/uploads/2017/08/YCDC-Guide-to-Talking-About-Dyslexia_Final032017.pdf

The book *Overcoming Dyslexi*a gives a broad overview of dyslexia with many practical implications and considerations. This book is often recommended to individuals who are new to learning about dyslexia or parents who are trying to navigate a new diagnosis. The other textbook, *Multisensory Teaching of Basic Language Skills*, is a textbook that goes in-depth into research in the field and understanding of key principles of literacy development and Structured Literacy instruction. This textbook was chosen by the university as it was decided to try this textbook in both the inaugural dyslexia course as well as integrating the textbook into other literacy courses.

This undergraduate course was delivered in a hybrid format with face-to-face meetings coupled with asynchronous learning weeks. The instructors initially agreed upon this format as the time together is valuable, but the syllabus was also heavy on reading material for participants. The asynchronous format allowed students to self-pace their learning, engaging with readings and instructional videos on their own schedules. The key topics and assignments from the syllabus are listed in Table 1 with descriptions to follow. The course topics align with the requirements listed out in Act 607. Topics aligned with Act 607’s requirements and were refined through faculty collaboration and feedback from state symposia.

**Table 1:** Course topics and major assignments

Course topic	Description	Syllabus assignments
**Overview of dyslexia:** Historical roots of dyslexia Present day findings on dyslexia Defining and Identifying the risk of dyslexia	Overview of the body of scientific work regarding dyslexia, including the history, epidemiology, and clinical presentation, including early clinical indicators of dyslexia and common, persistent classroom presentation	Assigned readings Discussion board Final exam Article 2 summary
Evidence-based practices	Overview of evidence-based instruction for individuals with dyslexia	Article 1 summary Discussion board Case study
Remediation	Remediation of weaknesses, fortification of strengths, and common accommodations to help mediate between the two	Assigned readings Final exam Case study
Dyslexia practitioner	Introduction to the process of becoming a dyslexia practitioner or dyslexia therapist	In class discussion

#### Overview of dyslexia

The *Overcoming Dyslexia* book was utilized to discuss the historical roots of dyslexia to present-day findings. Additionally, research from the Birsh textbook was also discussed in the classroom lecture. Additional reading and reflections allowed for new learning and reflections from the students. The final reflection required students to give detailed descriptions of defining and identifying dyslexia in the classroom.

#### Evidence-based practices

A case study included in the *Overcoming Dyslexia* book was utilized to practice identifying red flags of dyslexia during class. The Birsh textbook was used in lectures to discuss components of Structured Literacy instruction. Class time was spent reading, discussing, and practicing with instructional scripts or videos.

#### Remediation

The article summary gave students an opportunity to discuss remediation recommendations based on readings and class discussions. Videos were shared to show examples of dyslexia intervention and classroom accommodations during class discussions.

#### Dyslexia practitioner

As required by Act 607, the instructors discussed the pathways to becoming a dyslexia therapist or practitioner as laid out by the Louisiana Department of Education.

### Data collection

As the course began, instructors collected data in multiple ways to document the course implementation and outcomes. Data were collected from a variety of sources to provide a comprehensive understanding of the course’s implementation and impact. The students in each section completed six reflections throughout the course. An initial reflection was completed at the beginning of the course and a final reflection was written at the end of the semester. Additionally, reflections were submitted after major assignments to glean from the students’ perspectives. Two reflections were completed as discussion boards that allowed students to gain insight on unique perspectives and learnings from each other. These reflections were gathered throughout the course from students to capture their insights, learning experiences, and perceptions of the course’s relevance. Reflection prompts can be found in the Appendix. Pre- and postsurveys were administered at the beginning and end of the course to gather feedback on its effectiveness, including what aspects worked well and what needed improvement. The instructors recorded analytic notes in reflective journals to document their observations, challenges, surprises, and successes throughout the course. Additionally, the course syllabus, assignments, and instructional materials were analyzed to understand the course’s alignment with its intended objectives.

### Data analysis

To investigate the successes and challenges discovered by instructors while designing and implementing a state-mandated dyslexia course for students enrolled in Louisiana teacher preparation programs, the researchers analyzed the instructors’ analytic notes of observations and interactions, instructors’ weekly reflections, and student reflections regarding the course syllabus, course materials, course pacing, course content, and student learning outcomes. These notes of observations and interactions during class and in communication with students before, between, and after class meetings recorded critical instances where the instructors documented observed successes and challenges related to the implementation of the new dyslexia course. Next, we investigated our modifications, deletions, adjustments, and extensions made to the syllabus, course schedule, and the course instructional materials (used or not used with the students) and student reflections. Multiple readings of the notes and reflections were conducted to identify patterns and themes in the data related to successes, surprises, and barriers.

To determine how participation in this undergraduate dyslexia course impacted preservice teachers’ knowledge of dyslexia, we examined data from the students’ pre- and postsurveys and student reflections. For this analysis, we used both quantitative and qualitative methods to investigate the student outcomes. Quantitative results from the survey analysis are presented in Table 5.

### Ethical considerations

The study adhered to ethical guidelines, ensuring confidentiality and anonymity for all participants. Institutional Review Board (IRB) approval was obtained before conducting the study, and all data were securely stored to protect participant privacy.

Our research team consisted of literacy education professors and instructors with different experiences and positions within the university’s teacher preparation programs. This allowed for multiple insights into the formation and structure of the course. The two instructors in the course have extensive experience as dyslexia therapists, which allowed for a thorough understanding of what students should learn throughout the course and of how to teach adult learners. While the background knowledge of dyslexia assisted in the research for this case study, the instructors also acknowledge the potential bias that their lens may create in the analysis of the findings.

### Success: social–emotional impact and connection to individuals with dyslexia

As adult learners learn best when they can connect their learning to real-world experiences, class members with experience related to dyslexia (either themselves or a friend or family member) were able to share examples of how dyslexia impacts an individual. While the social–emotional impact of dyslexia was not something outlined in the course syllabus, this became a foundational component of the course that seemed to really motivate students to dive into their learning around dyslexia.

Students, as well as instructors, were able to discuss their experiences of knowing family or friends who struggled with reading. The discussion of the social–emotional impact inside and outside of the classroom was eye-opening for many students. During a classroom discussion in Week 1, the examples below record students’ comments that reflected their personal connections.

Kirby: I always wondered why other students got pulled out of class, but no one told me.

Sarah: I was so frustrated tutoring my first dyslexic child. I would get so mad at him because I didn’t understand what was happening.

Tina: I remember my dad struggled to read a book to me in elementary school and finally connecting the dots to his reading disability.

Elliot: Growing up, my dad had dyslexia. School was always really hard for him, and he still does not like to read out loud to this day. I feel like I understand a lot about dyslexia, growing up with a dad who is dyslexic.

One student (Hailey) told a story about not understanding the weight of dyslexia until college, when her good friend refused to play Scrabble with her and later disclosed how his reading disability continues to make spelling difficult for him.

Connecting to these experiences gave everyone in the class a feeling of responsibility toward understanding their role in dyslexia advocacy as a future classroom teacher. The instructors found that the initial in-person session was successful in creating a classroom of future teachers who felt a strong desire to advocate for their students with dyslexia. This opportunity to connect with each other and real-world experiences kept classroom engagement high during class meetings. Instructors both agreed that this was an imperative objective of the new course, although it was not explicitly laid out in the syllabus. This led to adaptations to course assignments to extend on the conversations and questions that arose from the first day of teaching and the evaluation of initial reflections.

### Success: modifications for real-world connections

Both instructors saw opportunities to extend the initial conversations and questions about dyslexia by adapting the syllabus assignments. As the instructors pulled from their own knowledge and resources, some course assignments were edited.

Two additional assignments were created to allow students to learn more about the dyslexia topic of their choice. The first was a series of article summaries in which students chose an article from a list of recommendations. Second, there was an opportunity to present about dyslexia through a dyslexia presentation. This was done in a jigsaw method so that students could present on their topic of choice and then learn about other topics. Student groups were formed and a list of topics was given that included the social-emotional impacts of dyslexia, dyslexia in adulthood, talking to parents about dyslexia, a structured literacy overview, common myths and misconceptions around dyslexia, and early intervention for dyslexia. Each group chose a topic of interest for their presentation. Presentations could either be a one-page handout or a PowerPoint presentation. Students were also given a list of resources to use as references such as the International Dyslexia Association, their assigned text, the reading league, and the Dyslexia Alliance for Black Children. Presentations were graded on explanation of topic, recommendations and resources, clarity, and effort. Students worked outside of class during asynchronous weeks to prepare for their presentations.

These notable modifications to the syllabus enabled students to select topics from a curated list, conduct independent research, and present their findings in groups to their peers. The method encouraged students to share responsibility for learning and teaching, creating a collaborative classroom environment. Many students contributed personal experiences or insights from their educational or professional backgrounds, which enriched discussions. The instructor also noted that the assignment prompted many conversations that continued outside of class time. In one instructor’s reflection from Week 8, she recorded that two students approached the instructor after this class to ask questions about a parent meeting they joined during their practicum experience at a local elementary school. The meeting involved a concerned parent whose child had identified characteristics of dyslexia in their child. The students were eager to tell their stories and ask for the instructor’s recommendations. Creating the space for students to engage in self-directed learning (Knowels et al., 2014) by choosing a topic of interest to learn and present was a successful practice, as course participants had a variety of experiences with dyslexia.

Another modification was necessary when, in August, the Louisiana Department of Education released an overview of dyslexia called the *Guide to Dyslexia*. This is a comprehensive guide created by Louisiana’s Department of Education to help families and educators understand dyslexia and the framework in place to help students with or at risk of dyslexia. In response to the release of *A Guide to Dyslexia*, and seeing the necessity of preservice teachers understanding dyslexia in the classroom, the instructors introduced an assignment designed to familiarize students with the document and its implications for identifying and supporting students with dyslexia. Students went on a scavenger hunt to understand the legislation around dyslexia in the state of Louisiana and to review the next steps when dyslexia is suspected. The guide is a practical resource that preservice teachers can revisit when dyslexia is suspected in their classroom.

While this was not included in the initial assignments planned, it was a beneficial way to spend instructional time. Students learned valuable information about dyslexia and how it is addressed in the state of Louisiana. Screening and assessing for dyslexia is an important topic. The guide discusses fluency screeners to flag deficits associated with dyslexia, characteristics of dyslexia by grade level, and an explanation of the umbrella of specific language disorders (SLDs) in reading, which is the term likely to be seen on a diagnosis over the specific term “dyslexia” currently in Louisiana. The instructors were able to use their knowledge of these current policies in their surrounding school districts to walk students through fluency screeners that are the primary screener for dyslexia in the school system, where the undergraduate students are conducting their field practicum experience. One instructor noted that many students had questions about the fluency assessment, and, while students had learned about the assessment in other coursework, it was helpful to see how it can be used to look for risk of dyslexia. The course assignments and adaptations are listed below in Table 2. These notable modifications were successful adaptations to the course assignments.

**Table 2:** Course assignments and adaptations

Course topic	Adaptations and teacher notes
**Overview of dyslexia:** Historical roots of dyslexia Present day findings on dyslexia Identifying risk of dyslexia	TED talks and other videos were shown around the brain research as it was a key interest to students and addressed many initial questions A *Guide to Dyslexia* (see below) also addressed many of these topics Final exam became overview of *Overcoming Dyslexia* book to ensure chapter readings, understandings, and reflections Dyslexia presentation was adapted for self directed learning to engage more in-depth with topic of choice around dyslexia Article summaries were adapted to include topics of choice with key research around dyslexia
Evidence-based practices	A *Guide to Dyslexia* in Louisiana was released and added as a necessary course component. An assignment was added to be sure students understand dyslexia in the classroom context Research vs. evidence-based learning article was added and discussed Dyslexia presentation was adapted for self directed learning to engage more in-depth with topic of choice around dyslexia
Remediation	Reading Rockets Reading 101 self-paced modules were added for students missing foundational knowledge of structured literacy More difficult to show inside the classroom Using group work discussions for case studies and having students draft an at-risk indicator checklist was helpful for students with placements at current schools. This helped them connect a real world experience with the content Video example of dyslexia intervention was shown for demonstration and well received by students Dyslexia presentation was adapted for self directed learning to engage more in-depth with topic of choice around dyslexia
Dyslexia practitioner	Class discussion and websites shared Video by a professional credentialing organization geared towards Universities was shown State level training opportunities such as dyslexia practitioner training cohorts along with the state level certification requirements were reviewed An article examining the benefits of adding a certified academic language therapist (CALT) to a dyslexia team was read and discussed

### Challenge: a siloed course

While the required dyslexia course content was identified in Act 206 (Louisiana Legislature, 2020), both instructors found it difficult to cover everything desired. There was little time to devote to classroom interventions and accommodations in the course readings and assignments. The course participants were completing other literacy courses simultaneously. However, there was little collaboration to weave dyslexia discussions into other coursework. While it was likely discussed in the other courses, the uncertainty made it difficult to be sure that all topics deemed worthwhile to cover in a teacher preparation program were appropriately addressed. Two examples from the instructors’ reflections and meetings were the dyslexia screeners and structured literacy. Table 3 lists the instructors’ concerns regarding dyslexia screeners and structured literacy.

**Table 3:** Challenges with dyslexia screeners and structured literacy content

Course topic	Challenges—instructor A	Challenges—instructor B
Dyslexia screeners	Email correspondence from other instructors mentioned discussing dyslexia screeners in the assessments course and a follow-up would have been helpful to coordinate the discussion around that in courses The *Guide to Dyslexia* was released close to the start of course which made planning difficult as syllabi across the department were likely done and distributed The White Paper about DIBELS and dyslexia was important to read over to be specific about the fluency red flags that point to dyslexia as mentioned in the *Guide to Dyslexia* published by the DOE Fluency is discussed in foundational literacy courses and assessments and this could be added there	The Louisiana Department of Education is still updating and deliberating resource guides concerning dyslexia. Since the course legislation passed, there was additional legislation passed concerning screening for dyslexia in schools. As development and guidance is ongoing, it makes it challenging to prevent the most relevant information regarding screeners Having a practicum or lab in alignment with this course would help with providing some hands-on experience for students The instructor found it challenging to relate what is happening in schools to the course. Many students in school placement situations mentioned that although they saw students being administered screenings such as DIBELS, dyslexia was never mentioned as part of the assessment process. Therefore the lack of school awareness on dyslexia can impact field placement experience
Structured literacy	Alignment with other literacy course content became problematic as the sequence of topics did not always match the outline from other courses While the Birsh textbook is an excellent resource, finding time to incorporate readings and practice from the textbook was limited	The instructor noted that this portion of the dyslexia course could be strengthened if it were cross connected with other literacy courses in the education department Students mentioned during the course module about fluency they had learned some things about fluency, but had not had as much exposure to structured literacy lessons in action Sample videos were helpful, and many students mentioned the desire to see more lessons being implemented as this wasn’t something they had broad exposure to in their field studies

As previously mentioned, course readings and materials were adapted throughout the semester. While the changes led to successful learning in the classroom, both instructors noted the difficulty of integrating the required materials into the course along with the many other popular resources for learning about dyslexia.

Instructors with greater knowledge and experience in dyslexia would be able to easily use the course syllabus as a foundation for teaching the course, with improvements to be made that allow room to implement other resources as they become available or in response to student interest and experiences.

### Challenge: classroom representation of dyslexia

Another challenge noted by instructors was difficulty providing classroom examples of dyslexia. While there was no field or practicum experience tied to this course, all students in the current two sections were simultaneously completing fieldwork for other education courses.

However, the second discussion board posted during an asynchronous week revealed that the students did not yet feel confident to report red flags of dyslexia from their classroom experiences. Students reflected that this may also stem from insufficient time in the class and revealed through the discussion board that teachers in their district no longer use small group instruction, so their opportunities to work with, or even listen to, students are limited.

This challenge is likely to continue and perhaps become more difficult in future sections as there will be diverse academic pathways for students needing this course: from elementary education to master’s students to secondary education students. In a postcourse discussion, instructors noted that this diversity in participation will require adjustments to ensure the course is relevant for all participants and suggested that the current syllabus is not a one-size-fits-all approach for appropriately addressing dyslexia.

It is important to note that this course was created as an introductory course to the topic of dyslexia. As students in this course will range from elementary teachers through secondary educators in a variety of content areas, dyslexia intervention practices and strategies are introduced but are not an in-depth focus of the course, which does impact students’ confidence in dyslexia understanding and intervention strategies. Providing websites with video examples of students receiving instruction (such as those mentioned in the Appendix) and a more extensive list of resources and examples can help students to better recognize red flags of dyslexia if they are not able to see real-world examples.

### Surprises during course implementation

After reviewing student reflections, one instructor noted the data revealed that misconceptions remain prevalent without explicit instruction of what dyslexia is as well as what dyslexia is not. The two misconceptions that were acknowledged most frequently in class reflections and discussion board posts were that dyslexia was a visual issue and that dyslexia is just seeing letters backwards. About 25% of students mentioned letter reversals in their initial reflections on dyslexia during Week 1 of the course. While students mentioned some misconceptions in the beginning of the semester assignments, students admitted to holding misconceptions throughout the semester in a variety of ways. Table 4 lists examples of student misconceptions.

**Table 4:** Evidence of student misconceptions

Misconception	Student responses
Dyslexia is a visual issue	“ I thought it was a vision issue vs. neurological.” (Tyler, presentation reflection) ”I chose common misconceptions about dyslexia because there was so many I experienced when first coming to this class. The first day of class, I remember being very shocked that dyslexia wasn’t in the eye, yet the brain.” (Austin, presentation reflection)
Dyslexia is just seeing letters backwards	“This isn't the first course that I've learned things about dyslexia but after my course about disabilities and learning disabilities, I thought that I had it all figured out.. I was wrong. I didn't know that people with dyslexia didn't see letters backwards at all... In reality, it's the manipulating and decoding that happens in the brain (not "visually") that affects the learner.” (Laura, discussion board) “Before this class, my understanding of dyslexia didn't expand beyond seeing the letters "d" and "b" and other things like this.” (Morgan, discussion board)

One instructor noted that misconceptions were even present in students with their one personal diagnosis of dyslexia, documenting that a student described to the class how the color yellow worked best for her dyslexia and colored paper was an accommodation she received for testing. Both instructors noted their surprise at the misconceptions that were discussed at the beginning of the course.

### Impact on preservice teachers’ knowledge of dyslexia

As previously mentioned, a pre- and postsurvey was distributed to course participants at the beginning and end of the semester. The survey was optional for students and distributed via an anonymous link. At the beginning of the semester, 29 precourse surveys were collected and 28 postcourse survey responses were collected at the end of the course. The survey measured three categories of knowledge: understanding of dyslexia, evidence-based practices for students with dyslexia, and professional pathways. Survey results revealed four self-reported questions with statistically significant differences in pre and postsurvey results: Question 1: I have a basic understanding of what dyslexia is, Question 3: I understand the differences between dyslexia and other learning disabilities, Question 5: I have previous experience or knowledge of evidence-based practices for teaching students with dyslexia, and Question 12: I am aware of the pathways to becoming a certified dyslexia specialist.

Data collection was analyzed from pre and postsurveys to determine if samples were normally distributed. As samples were determined not to be normally distributed, a two-sample *t*-test assuming unequal variances was conducted to compare participants’ self-reported knowledge of dyslexia before and after completing the course. Each question was then individually analyzed to pinpoint areas of greatest increase in self-reported knowledge. As shown in Table 5, results indicated a statistically significant increase in preservice teachers’ knowledge of dyslexia and other learning disabilities, evidence-based practices, and awareness of pathways to becoming a certified dyslexia specialist.

**Table 5:** Pre- and postsurvey results

Question	Presurvey data	Postsurvey data	Analysis
Q1. I have a basic understanding of what dyslexia is	*M* = 3.75, SD = 0.84	*M* = 4.44, SD = 0.58	*t*(48) = 3.57, *p* < 0.001
Q2. I am aware of the common signs and symptoms of dyslexia in students	*M* = 3.21, SD = 1.07	*M* = 4.41, SD = 0.57	*t*(42) = 5.19, *p* = 5.65
Q3. I understand the differences between dyslexia and other learning disabilities	*M *= 3.33, SD = 0.88	*M *= 4.04, SD = 0.59	*t*(45) = 3.46,* p* = 0.001
Q4. I am familiar with the role of structured literacy in supporting students with dyslexia	*M* = 2.68, SD = 1.02	*M* = 4.03, SD = 0.76	*t*(50) = 5.62,* p *= 8.57
Q5. I have previous experience or knowledge of evidence-based practices for teaching students with dyslexia	*M* = 2.25, SD = 1.12	*M* = 3.44, SD = 1.28	*t*(51) = 3.69, *p* < 0.001
Q6. I am familiar with the historical development of dyslexia as a recognized condition	*M* = 2.5, SD = 1.14	*M* = 3.67, SD = 0.83	*t*(49) = 4.35, *p* = 6.89
Q7. I understand the neurological basis of dyslexia and its impact on reading	*M* = 2.93, SD = 0.81	*M* = 4.19, SD = 0.56	*t*(48) = 6.71, *p *= 2.08
Q8. I can identify the early signs of dyslexia in students	*M* = 2.68, SD = 1.02	*M* = 4.04, SD = 0.76	*t*(50) = 5.62,* p *= 8.57
Q9. I am aware of the persistence of dyslexia symptoms into adolescence and adulthood	*M* = 3.18, SD = 1.09	*M* = 4.37, SD = 0.56	*t*(41) = 5.12, *p* = 7.74
Q10. I am knowledgeable about structured literacy and its importance in teaching students with dyslexia	*M* = 2.86, SD = 1.15	*M* = 4.15, SD = 0.72	*t*(46) = 5.03,* p* = 8.03
Q11. I understand the principles behind evidence-based instruction for dyslexia	*M *= 2.57, SD = 0.84	*M* = 4.15, SD = 0.66	*t*(51) = 7.79,* p* = 3.35
Q12. I am aware of the pathways to becoming a certified dyslexia specialist	*M *= 2.14, SD = 0.80	*M* = 3.07, SD = 0.92	*t*(52) = 1.67, *p* < 0.001
Q13. I understand the ethical responsibilities of professionals working with dyslexic students	*M* = 3.82, SD = 0.94	*M* = 4.0, SD = 0.92	*t*(53) = 0.71, *p* = 0.48

When analyzing responses to individual postsurvey items, 86% of participants agree or strongly agree that they understand the difference between dyslexia and other learning disabilities, compared to only 38% of participants at the beginning of the course. Additionally, 54% agree or strongly agree that they have knowledge of evidence-based practices for teaching students with dyslexia, an increase from the initial 14% of participants.

Data was also analyzed from initial and final course reflections. At the beginning of the dyslexia course, an initial reflection gave students an opportunity to express their experiences and questions about the topic of dyslexia. The data revealed that 75% of students expressed that they had little to no knowledge about dyslexia. For example, Cheryl reported, “I am not too familiar with dyslexia. I just know about them mixing up letters, and it’s harder to read.” Destin wrote, “The term is somewhat familiar to me; I know in broad terms what it is but not what it consists of.” This finding shows the need for the inclusion of foundational literacy knowledge and a clear definition of dyslexia at the beginning of this course.

The data also revealed that students’ “knowledge of dyslexia” mainly consisted of knowledge of the social and educational struggles that a person with dyslexia may experience, not an understanding of the definition of dyslexia or red flags that may be seen in the classroom. For example, Rosalind stated, “As far as dyslexia goes, my father and brother both have it. Seeing my young brother be held back in first grade, pulled for small groups, and gain an IEP truly made me feel sympathetic to those who did not have reading come to them like second nature.” Mary wrote, “Writing this has made me realize I know nothing about dyslexia, or my brother’s experience with it.” And Austin responded, “Dyslexia is a term I am familiar with. Not much, but a little bit. My best friend’s little brother has dyslexia, and I can remember school being hard for him.” This initial reflection provided a great springboard for the conversations and activities that followed throughout the semester.

At the end of the course, students noted their personal growth in knowledge around dyslexia from the course, as shown in Table 6.

**Table 6:** Students’ knowledge of dyslexia increased over time

Student	Response
Brooks	This topic has really opened my eyes to how much deeper this learning disorder goes beyond the surface of academic struggles
Sarah	My mindset has shifted and now I want to be sure to be vigilant in my classroom to look out for the signs in students because of the importance of early intervention. Now that I have a better understanding of phonics, phonological and phonemic awareness, I feel that I can identify when there is a discrepancy in the students’ learning
Allison	Before this course, my only knowledge of dyslexia was the Bella Thorne commercial from Disney Channel. I truly thought that it was only when someone would flip their letters when spelling or reading. I learned that many of the signs shown by students with dyslexia are associated with challenges in reading, spelling, and writing. There are many common misconceptions when it comes to dyslexia. People tend to believe many myths that are spoken about dyslexia and dyslexic behaviors. This is why it is important to research dyslexia instead of using what you think you know
Mia	My biggest take away from this class was learning more about dyslexia as a whole. Prior to the start of this course I really didn’t know much about Dyslexia
Emma	Biggest takeaway—learning exactly what dyslexia was and what are signs to look for when identifying students with dyslexia. Learning that it is way more common than I thought—one in five. I enjoyed listening and hearing from the guest speaker

Students expressed positive changes in their understanding of dyslexia and dyslexia instruction.

## Discussion

### Legislative catalyst

As previously mentioned, Act No. 607 (Louisiana Legislature, 2022) required teacher preparation programs to include a three-credit-hour course focused on teaching students with dyslexia beginning with the 2024–2025 academic year. This legislation regarding dyslexia instruction provided an opportunity for programs to lay the groundwork for the knowledge that educators need around the common reading deficits associated with dyslexia. Dyslexia is a common reading disorder that affects a student’s ability to read and spell, and classroom teachers need to be aware of what dyslexia is and the role in identifying, remediating, and supporting students with dyslexia. While it became evident that students became more comfortable and knowledgeable on the topic of dyslexia, there are several considerations that must be made as an inaugural course is launched and appropriately implemented. While the local legislation was a catalyst for the course, the positive response and knowledge gained by the students highlighted the importance of increasing dyslexia education in teacher preparation programs.

### Addressing misconceptions

The findings of this study agree with other research pointing out that misconceptions still remain among pre-service teachers (Pelteir, 2020; Washburn et al., 2016) concerning seeing letters backwards and that dyslexia stems from visual issues. Participants in this case study were able to shift their mindset around these misconceptions with explicit instruction and discussion.

As the dyslexia course was developed to satisfy the needs of the university and the legislature, several considerations needed to be made around syllabus development, instructors, and course integrations into degree plans. At the close of the course, the instructors debriefed to develop guiding considerations for institutes who wish to implement a course focused on deepening students’ understanding of dyslexia in a teacher preparation program.While the initial syllabus was helpful in meeting the required objectives of the course, adjustments were made to create more relevant application and understanding around dyslexia and opportunities for self-directed learning, as suggested by Knowles and colleagues’ (2014) theory around working with adult learners. These adjustments were successful in helping students to gain foundational knowledge around dyslexia.

Findings showed it is imperative that the instructors are not only knowledgeable about dyslexia but are also knowledgeable about the current state of dyslexia in state and in classrooms. Instructors must find ways to connect the discussion of dyslexia back to the classroom. Some considerations may be video examples of dyslexia and dyslexia intervention and/or school visits.

### Considerations for teacher preparation programs

Many topics that are imperative to understanding the reading deficits associated with dyslexia fit into a broader context of literacy education and teacher development. A key finding from this research was the need for further collaboration and alignment across literacy courses and instructors. Ensuring that dyslexia discussions are incorporated into foundational literacy courses can help undergraduate students see the connection between their literacy courses, fieldwork, and the dyslexia course. Teacher preparation courses are too often siloed instead of strategically designed to weave together the knowledge important for understanding literacy development and deficits.

### Limitations and indications for future research

While this specific course was developed in response to specific laws in Louisiana, which limit the generalizability of transfer, several elements of this course could be embedded within overall literacy instruction with the intent of graduating educators who have knowledge of the signs and symptoms of dyslexia. Our findings revealed that many preservice teachers hold misconceptions about dyslexia, so it is important that teacher preparation programs intentionally plan for instruction and discussion focused around dyslexia.

## Conclusion

As numerous teacher preparation programs nationwide work to adapt and revitalize literacy instruction, the implications of this research are far-reaching. This study detailed how the legislative mandates were put into action through the course creation and instruction and provides practical considerations for other states and universities. Recommendations for improving dyslexia education in preservice teacher programs, based on the instructors’ experiences and the course outcomes, benefit the academic community and stakeholders in the field of education. While this study centered around a stand-alone course for dyslexia, it is important to note that these topics can, and should, be integrated into existing literacy courses. Institutions seeking to incorporate dyslexia education into their coursework and programs, either through a stand-alone course or integrated into other courses, are encouraged to collaborate through professional development and/or course design workshops focused on course alignment around assessment, tiers of instruction, and reading disorders such as dyslexia.

## Appendix. A resource guide: recommended resources, readings, and assignments for a dyslexia course in teacher preparation programs

 This appendix provides a curated list of recommended readings, instructional resources, and assignment ideas for university-level courses designed to prepare preservice teachers to support students with dyslexia. These materials are grounded in current research and were piloted or discussed during the implementation of Louisiana’s Act 607 dyslexia course.

### Recommended textbooks

- Birsh, J. R., & Carreker, S. (Eds.). (2018). Multisensory teaching of basic language skills (4th ed.). Brookes Publishing.
  *While this is an excellent resource, it is expensive and would best be utilized over multiple literacy courses.
- Shaywitz, S. E., & Shaywitz, J. (2020). Overcoming dyslexia (2nd ed.) Vintage.

### Recommended assignments

#### Reflection questions

Reflection questions can be used throughout to gauge knowledge and understanding of desired topics. Suggested reflection questions are as follows:

- In this initial reflection, please provide a detailed description of who you are, including your teaching goals, your journey into education, and any prior knowledge or experiences you have related to dyslexia. You may choose to respond in separate sections or weave your responses into a cohesive narrative, using the guiding questions below; your reflection should be approximately one full page or1 to 22 well-developed paragraphs per section.
- Reflect on how this understanding of reading as a learned skill challenges the assumption that reading should come easily to everyone. How should this influence the way reading is taught in schools?
- After reading about the common misconceptions of dyslexia, were there any that you initially held? What was most impactful about learning about your brain as it learns to read?
- What responsibilities do you have as a future educator for supporting your students with characteristics of dyslexia?
- As we close out the semester, reflect on your learning. How would you define dyslexia in your own terms and how can you support students with dyslexia in your future classroom?
- What is one takeaway you gained from the class presentations? What questions do you still have?
- After completing the book Overcoming Dyslexia, complete this reflection. Shaywitz emphasizes that dyslexia is a “hidden disability.” What does that mean, and why is it important for teachers to understand? Shaywitz discusses the importance of early identification and intervention. How might this influence your future classroom decisions?

### Article summaries

Students can be assigned specific articles or videos to review and summarize, reflect, and connect to classroom implications. Individual articles can be assigned, or an assignment could reflect an annotated bibliography of multiple readings over the semester.

### Case study analysis

Provide a student profile and ask students to analyze risk indicators, suggest assessment approaches, and propose classroom supports.

### Jigsaw group presentation

Students choose dyslexia-related topics (e.g., structured literacy, myths, adult dyslexia, and social–emotional impacts) and present via a handout or PowerPoint using approved resources. Presentations are done in class to their peers. See suggested websites and videos for recommended resources that could be utilized in this activity.

### Dyslexia scavenger hunt

Use your state’s Department of Education website or similar document. Create a scavenger hunt activity focused on legislation, screening tools, accommodations, and response pathways for an interactive activity that empowers students to understand next steps when dyslexia is suspected in their classroom.

### Structured literacy overview

Discuss common reading deficits associated with dyslexia and show examples of intervention and classroom instruction. This can be from textbooks such as the Birsh book mentioned above or through recommended websites.

### Book reading and discussion

*Overcoming Dyslexia* (or book of choice) can be read throughout the semester with chapter quizzes, reflections, and discussions to guide conversations around dyslexia.

### Final exam or capstone reflection

Assess understanding of key course concepts from readings, discussions, and practical assignments.

### Field placement assignments

While our course was not directly tied to a field placement, assignments could easily be created to connect dyslexia education to classroom experiences. Suggestions include teacher interview, classroom observations and reflections, and observing/working with a student with characteristics of dyslexia.

## Recommended readings and resources

- SFerrer, E., Shaywitz, B. A., Holahan, J. M., Marchione, K. E., Michaels, R., & Shaywitz, S. E. (2015). Achievement gap in reading is present as early as first grade and persists through adolescence. Journal of Pediatrics, 167(5), 1121–1125. https://doi.org/10.1016/j.jpeds.2015.07.045
- SThe Yale Center for Dyslexia and Creativity. (n.d.). Speaking with one voice: A guide to talking about dyslexia. https://dyslexia.yale.edu/wp-content/uploads/2017/08/YCDCGuide-to-Talking-About-Dyslexia_Final032017.pdf
- Accommodating Dyslexia in a Classroom Setting- Reading Rockets https://www.readingrockets.org/topics/dyslexia/articles/accommodating-studentsdyslexia-all-classroom-settings
- Odegard, T.N. & Dye, M. (2024).The Gift of Dyslexia What harm is it? Annals of Dyslexia, 74. 143-157. https://doi.org/10.1007/s11881-024-00308-9
- The Reading League. The Science of Reading: A Defining Movement. https://www.thereadingleague.org/what-is-the-science-of-reading/
- Video Resources: Reading Universe https://readinguniverse.org/explore-teaching-topics Reading Rockets https://www.readingrockets.org/
- Louisiana Department of Education. A Guide to Dyslexia. https://doe.louisiana.gov/docs/default-source/academics/a-guide-to-dyslexia-inlouisiana. pdf

*Breaking down and explaining the state’s requirements and language around dyslexia is important to ensure our preservice teachers know how to navigate the signs of dyslexia in their future classroom. While the Guide to Dyslexia from the Louisiana Department of Education was beneficial for us, we recommend checking out your state’s website.
